# Retraction Note: Xia-yu-xue decoction (XYXD) reduces carbon tetrachloride (CCl4)-induced liver fibrosis through inhibition hepatic stellate cell activation by targeting NF-κB and TGF-β1 signaling pathways

**DOI:** 10.1186/s12906-022-03714-x

**Published:** 2022-09-06

**Authors:** Cheng Liu, Xia Yuan, Le Tao, Zhuoan Cheng, Xiuqin Dai, Xia Sheng, Dongying Xue

**Affiliations:** 1 Experimental Research Center, Putuo Hospital, Shanghai University of Traditional Chinese Medicine, Shanghai, 200062 China; 2 Department of Pharmacy, Putuo Hospital, Shanghai University of Traditional Chinese Medicine, Shanghai, 200062 China; 3 Department of Infectious Disease, Putuo Hospital, Shanghai University of Traditional Chinese Medicine, Shanghai, 200062 China; 4 Department of Pathology, Putuo Hospital, Shanghai University of Traditional Chinese Medicine, Shanghai, 200062 China

**Retraction Note: BMC Complement Altern Med 15, 201 (2015)**

**https://doi.org/10.1186/s12906-015-0733-1**

The Editor has retracted this article. After publication, concerns were raised regarding western blot image similarities between Fig. 3B in this article and Fig. 3C in another article with one shared author [1] that was under consideration within a close time frame.

Additionally, the JNK blots in Fig. 3B are presented upside-down, and there appear to be unexplained breaks in the western blot backgrounds in Fig. 4B.

The authors have been unable to provide the original blots from the experiments reported in this article. The Editor therefore no longer has confidence in the presented data.

Author Zhuoan Cheng agrees to this retraction. Author Cheng Liu has not explicitly stated whether they agree to this retraction notice. Authors Xia Yuan, Le Tao, Zhuoan Cheng, Xiuqin Dai, Xia Sheng and Dongying Xue have not responded to any correspondence from the editor or publisher about this retraction.
